# Analytical Validation of Loss of Heterozygosity and Mutation Detection in Pancreatic Fine-Needle Aspirates by Capillary Electrophoresis and Sanger Sequencing

**DOI:** 10.3390/diagnostics14050514

**Published:** 2024-02-28

**Authors:** Venkata Arun Timmaraju, Sydney David Finkelstein, Jonathan Adam Levine

**Affiliations:** Interpace Diagnostics LLC, Pittsburgh, PA 15222, USA; sfinkelstein@interpace.com (S.D.F.); jlevine@interpace.com (J.A.L.)

**Keywords:** loss of heterozygosity (LOH), pancreatic cancer, allelic imbalance, sanger sequencing, capillary electrophoresis (CE), next-generation sequencing (NGS), long-read sequencing

## Abstract

Pancreatic cystic disease, including duct dilation, represents precursor states towards the development of pancreatic cancer, a form of malignancy with relatively low incidence but high mortality. While most of these cysts (>85%) are benign, the remainder can progress over time, leading to malignant transformation, invasion, and metastasis. Cytologic diagnosis is challenging, limited by the paucity or complete absence of cells representative of cystic lesions and fibrosis. Molecular analysis of fluids collected from endoscopic-guided fine-needle aspiration of pancreatic cysts and dilated duct lesions can be used to evaluate the risk of progression to malignancy. The basis for the enhanced diagnostic utility of molecular approaches is the ability to interrogate cell-free nucleic acid of the cyst/duct and/or extracellular fluid. The allelic imbalances at tumor suppressor loci and the selective oncogenic drivers are used clinically to help differentiate benign stable pancreatic cysts from those progressing toward high-grade dysplasia. Methods are discussed and used to determine the efficacy for diagnostic implementation. Here, we report the analytical validation of methods to detect causally associated molecular changes integral to the pathogenesis of pancreatic cancer from pancreatic cyst fluids.

## 1. Introduction

Pancreatic cancer (PC) has the third-highest mortality rate amongst malignancies in the United States, with the expectation to be ranked second by 2030 [1]. Pancreatic carcinogenesis represents a complex, multistep process resulting in a high frequency of precancerous lesions. Pancreatic cancer can arise from solid masses or pancreatic cyst precursors. These lesions may either remain stable or undergo further neoplastic progression with an overall low incidence of malignant transformation but a high cancer mortality rate even when cancer is detected at an early stage [2,3]. Currently, neoadjuvant and adjuvant therapies do not yield optimal outcomes, nor does the combination of chemoradiotherapy [4]. Fully developed, PC has a five-year survival of only 12%, accentuating the need for better diagnostic tests and treatment modalities [5].

Multimodality therapy that includes surgery offers the only prospect of a cure for PC. Surgery is highly invasive and associated with a mortality rate of 3–7% [6,7,8]. The recovery time is long and complex, resulting in increased morbidity and a significant monetary burden on the patient and the medical system [9,10]. However, alternative approaches such as chemotherapy, radiation therapy, or cyst ablation by chemical instillation have yet to prove effective [11]. Management can be challenging, as patient morbidity and mortality are significant considerations for this relatively costly intervention, as is patient quality of life; however, the aggressive pathogenicity of PC is even more challenging to manage. Balancing these decision-making factors continues to result in surgical pancreatectomy for benign disease in ~80% of cases as determined by surgical pathology [12]. Thus, there is a vital need for preoperative testing that can more accurately discriminate benign, stable cystic disease from malignant or high-grade dysplastic cystic disease at high risk for progression to cancer [12,13,14].

Pancreatic adenocarcinomas (Pas) account for the majority of PCs. A large number of Pas originate within the epithelial lining of the pancreatic duct system [15]. These lesions are characterized by the formation of solid masses and/or pancreatic cysts, both of which can be precursors that are detectable by medical imaging and amenable to endoscopic sampling [16]. Distinguishing between patients with a stable precursor phenotype that can be followed versus patients with a precursor lesion who will benefit from surgical intervention is a continued need in the medical community, as most pancreatic cysts (>85%) are benign [17,18]. Choosing the optimal biomarkers requires an understanding of PC oncogenesis and is key for a successful assay design to diagnose a disease with a high incidence of precursors and a low incidence of invasive disease with a high mortality rate.

Pancreatic cysts are primarily detected through incidental findings; further imaging can help elucidate the cyst type and pathogenicity. Computer Tomography (CT) and Magnetic Resonance Imaging (MRI) are 40–60% accurate in predicting the histologic diagnosis and 70–90% in differentiating non-aggressive versus aggressive lesions. One of the top imaging modalities is Endoscopic Ultrasound (EUS). EUS has a broad range of detecting mucinous lesions, with specificities and sensitives reported from 56–78% to 45–67%. Also, EUS has a 75% sensitivity and 83% specificity for high-risk lesions [19,20,21,22,23].

Cysts can be of several types, including, but not limited to, pseudocysts, Serous Cyst Adenomas (SCAs), and mucinous cysts such as IPMNs (Intraductal Papillary Mucinous Neoplasms) and MCNs (Mucinous Cystic Neoplasms). Mucinous cysts (~15–30%) have the potential for malignant transformation to PDAC, while a Serous Cyst Adenoma (SCA) is primarily benign. However, 16% of resected pancreatic cysts are SCAs. The most recent guidelines stipulate that *VHL* mutations can help differentiate a SCA from a mucinous cyst from a SCA and demonstrate the sensitivity and specificity of *KRAS* and *GNAS* with mucin, illustrating the utility of a genetic analysis. Further, the guidelines directly state “*GNAS* mutation at codon 201 is exclusively observed in IPMN, and assessment of *GNAS* mutation is useful to discriminate IPMN from MCN”. Lastly, a pseudocyst, benign by its namesake, can be diagnosed by the absence of *KRAS* and *GNAS* mutations complemented by a high amylase level, which can help rule in a pseudocyst [24,25,26].

Pancreatic tumorigenesis involves acquiring oncogenic mutations and altered tumor suppressor gene function. These genetic alterations enhance the proliferation of ductal lining cells leading to PC precursors [15] and detecting such alterations can influence intervention and/or surveillance frequency [27,28]. Mutations in hotspot regions of codons 12 and 13 in the *KRAS* gene and codon 201 in the *GNAS* gene are well-recognized as oncogenic drivers, often present in the samples at high variant allele frequency (VAF) and are commonly acquired early in the multistep PC tumorigenesis process [29]. Such changes identify individuals who have developed pancreatic ductal lining cell neoplasia; however, these oncogene alterations can be found in both non-progressing and progressing patients [30]. A strong correlation exists between *KRAS* and *GNAS* oncogene mutation and tumor suppressor gene loss, with mucinous cyst transformation taking the form of IPMNs and MCNs [29,31]. Of potentially greater importance, continued progression involves tumor suppressor loss, which can involve virtually all chromosomes with a well-characterized imbalance of genomic loci, including 1p, 3p, 5q, 9p, 10q, 17p, 17q, 18q, 21q, and 22q, as well as other regions [30,32,33,34].

These well-recognized forms of mucinous cystic disease manifest in a spectrum ranging from benign, nonprogressive lesions to malignant. The progression to malignancy correlates with the continued accumulation of mutations, especially tumor suppressor gene loss [35]. Losing one of the two tumor suppressor gene copies represents the second step of the Knudson two-hit hypothesis that has been established in many forms of cancer, including PC [36,37]. The first of the two steps takes the form of either sequence mutation or other forms of genomic alterations, resulting in the inactivation of one of the two normal copies of an affected tumor suppressor gene [38,39]. The second step, losing the remaining normal copy, is critical in inactivating the tumor suppressor gene’s function, resulting in a complete loss of suppressor function. Targeting detection of the second step, loss of the normal copy (or allele), is valuable in establishing causality, as genomic deletions tend to be relatively large-sized and encompass chromosomal regions where multiple tumor suppressor genes may reside [38,39]. Hence, detecting the second step, or the loss of the second allelic copy, provides the greater diagnostic value and causal association with neoplastic progression. This loss is referred to as the loss of heterozygosity (LOH) and is characterized by an allelic imbalance at highly polymorphic short tandem repeat (STR) loci [40,41,42,43,44]. Polymorphic STRs vary greatly in size and are unknown before analysis. The difference can be one repeat or up to twenty-five repeats, creating a moving target the size of the STRs for detection [45]. In addition, expansion alterations add a further complication in STR detection [46]. Multiple methodologies are available for nucleic acid-based detection and measurement. Therefore, selection of the instrumentation is vital to the reproducibility and accuracy. In this study we compare methods that analyze nucleic acid sizing and sequencing.

Electrophoresis utilizes the electrochemical potential to allow the migration of molecules such as DNA, RNA, or proteins through a gel gradient. The variables include voltage, wattage, length of the gel, and the percentage of the gel as the separating agent, such as acrylamide or agarose [47]. This technology dates to the 1930s and has continued to evolve over the past 90 years but continues to be a mainstay in the community. When a biological analyte such as DNA is applied to electrophoresis, DNA resolution occurs based on the analytes’ electrical potential. In this study, denaturing electrophoresis will be employed instead of native electrophoresis, as native separation is based on size/length and shape, not just size/length [48]. Capillary electrophoresis (CE) was initially developed in the 1980s and “became the method of choice for fast high-resolution DNA sequencing in the nineties of the last century” [47]. Sanger sequencing, invented in the late 1970s by Frederick Sanger, utilizes electrophoresis and migrated to capillary electrophoresis, becoming the gold standard for DNA sequencing. Sanger sequencing incorporates a chain-terminating labeled dideoxynucleotide to amplicons of the specific region, and the DNA fragments are resolved by electrophoresis [49,50].

Newer techniques have since emerged and evolved, such as next-generation sequencing (NGS), or short-read sequencing, and the newer third-generation (TGS), or long-read sequencing. All three methods (Sanger sequencing, NGS, and TGS) have been used in several aspects of nucleic acid analysis. NGS analyzes short DNA fragments and then reassembles the individual reads by mapping them to a reference genome. For NGS analysis, sample libraries are prepared with small, fragmented DNA followed by sequence detection. The detection mechanisms vary by the platform used, such as fluorescence (Sequencing by Sequencing (SBS)/Illumina), Nanoball technology (Complete Genomics/BGI), or pH changes (Thermo Fisher Semiconductor Sequencing) [51,52]. Although NGS has many advantages, sequencing of repetitive regions is complicated. The size and length of the repeats, and the potential for repeat expansion, pose a technical challenge irrelevant of the NGS chemistry (SBS, Nanoball, or Semiconductor). A downstream analysis can also be complicated by artifacts of alignment and assembly of STRs owing to their highly repetitive nature [53]. Third-generation sequencing, or long-read sequencing, does not have this limitation because the sequenced DNA fragments are longer, allowing easier alignment and assembly of sequences. Long-read sequencing can use either fluorescent detection (Pac Bio Single Molecule Real-Time (SMRT)) or electrical potential (Oxford Nanopore). Both have been decreasing in cost and increasing in accuracy over the years, yet both are still more expensive than short-read sequencing and, in some cases, depending on the sequence, less accurate for single-nucleotide polymorphism (SNP) resolution [54,55]. However, when it comes to scaling for SNP detection, NGS and TGS are cost-prohibitive when only a few SNPs need to be assessed but are cost-effective with larger marker panels for comprehensive profiling.

The purpose of this study is to evaluate the capability, accuracy, and precision of CE and Sanger sequencing compared to the newer technologies (NGS and TGS) for continued use as the optimal diagnostic methods for STRs and a low number of SNPs. We report the design of an assay for the accurate detection of two molecular changes: (1) loss of heterozygosity (LOH) in tumor suppressor gene loci by analyzing highly polymorphic microsatellite regions comprising tetranucleotide short tandem repeats (STRs) using capillary electrophoresis, and (2) the presence of single-nucleotide mutations by using Sanger sequencing in cell-free deoxyribonucleic acids (DNAs) extracted from pancreatic fine-needle aspirates. This assay is designed to serve as a reliable biomarker of nonprogressive versus progressive PC-associated precancerous lesions to optimize the decision-making for surveillance and timing of the surgical intervention.

## 2. Materials and Methods

### 2.1. Samples

Clinical samples were collected as a part of routine testing by the PancraGEN^®^ (pancreatic cancer risk classifier) test at Interpace Diagnostics. Samples were analyzed in this study in a retrospective manner approved by the Advarra Institutional Review Board (approval number Pro00074090). Experiments were performed under the College of American Pathologists (CAP)/Clinical Laboratory Improvements Amendments (CLIA) guidelines. Positive and negative control samples for the LOH analysis were created from DNA extracted from healthy blood donors. For *KRAS* and *GNAS* mutation detection, Horizon controls HD289 reference standard and DNA extracted from HD104-083 (Horizon Discovery, Cambridge, UK) respectively, were used as controls.

For the orthogonal analysis of LOH detection, 17 positive control samples were analyzed by CE and TGS. For the orthogonal analysis of mutation detection, 106 samples (50 negative and 56 positive) for *KRAS* or *GNAS* mutations were analyzed by Sanger sequencing and NGS.

For accuracy of the LOH detection, residual DNA extracted from 203 pancreatic cyst fluid samples and their matching buccal swabs collected during routine clinical testing at the Interpace Diagnostics laboratory (Pittsburgh, PA, USA) were analyzed by CE on 2 instruments. For accuracy of the mutation detection, residual DNA from 30 clinical samples for each *KRAS* and *GNAS* (15 negative and 15 positive) were analyzed by Sanger sequencing on 3 sequencing instruments.

For reproducibility of the LOH analysis, 10 sample pairs (positive pancreatic cyst and matching normal samples) were analyzed 8 independent times. For reproducibility of the Sanger sequencing, 3 pancreatic cyst fluid samples positive for *KRAS*, and *GNAS* mutation, respectively, were analyzed in triplicate on 3 different instruments (*n* = 9). Reproducibility in a clinical setting was also demonstrated by analysis of the process controls from one month of testing data. This includes LOH detection from a positive control sample and mutation detection from Horizon controls (HD104-083 and HD289).

LOH limit of the detection analysis was carried out by mixing two samples homozygous for the *D5S615* STR to create a range of samples reflecting allelic imbalance, followed by CE analysis (*n* = 23). SNP limit of detection was carried out by diluting a mutation-positive into a mutation-negative sample to create samples across a range of % VAF followed by Sanger sequencing (*n* = 21). For detection at low input by CE, a LOH-positive clinical sample and its matching normal were analyzed across a range of input concentrations (2.5 ng to 0.156 ng). For detection at low input by Sanger sequencing, a *KRAS* positive sample was analyzed across a range of input concentrations (10 ng to 0.25 ng).

### 2.2. DNA Extraction

DNA was extracted using the QIAamp DNA Blood Mini kit 250 (Qiagen, Hilden, Germany) according to the manufacturer’s instructions on the QIACube automated nucleic acid extraction system (Qiagen). Samples were quantified by Nanodrop spectrophotometry or Qubit DNA analysis (Thermo Fisher Scientific, Waltham, MA, USA).

### 2.3. Loss of Heterozygosity (LOH) Analysis by Capillary Electrophoresis

**Figure 1:**
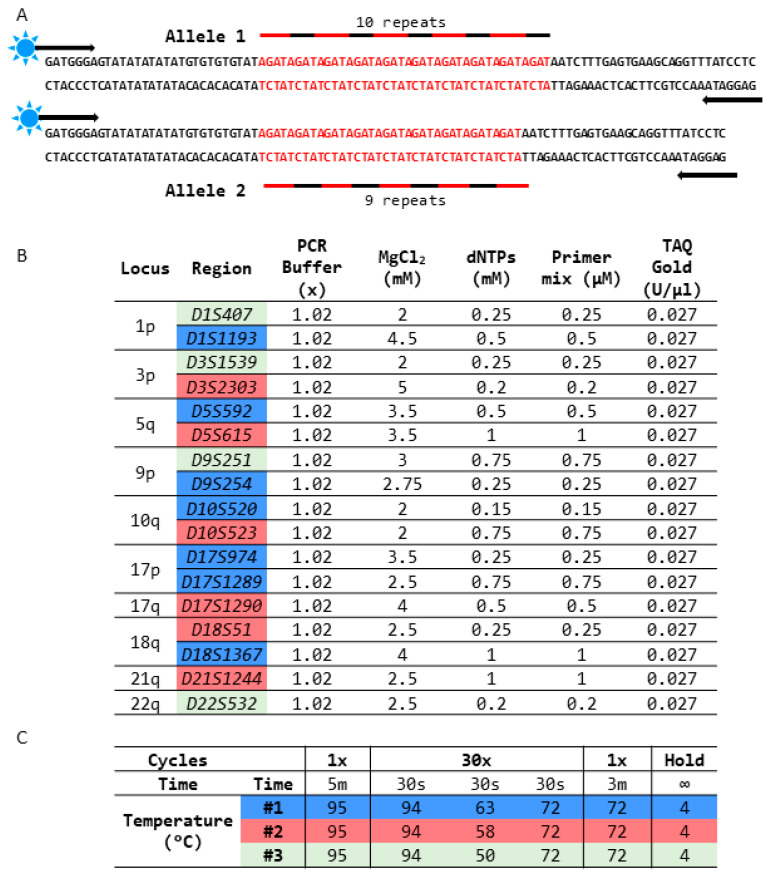
Design strategy of LOH detection. (**A**) Representative design shows a sample heterozygous for the *D9S254* STR region containing 9 and 10 AGAT repeats. Each STR region is detected by a specific primer pair, where one of the primers is fluorescently tagged and used for detection during capillary electrophoresis. (**B**) LOH panel markers were analyzed with specific PCR conditions (#1—blue, #2—red, and #3—green) with marker-specific MgCl_2_ and dNTP concentrations. (**C**) The 3 PCR conditions used for amplification of the LOH loci are listed and differ in PCR primer annealing temperature. The arrows in the figure indicate direction of primer binding.

For LOH analysis, a specific polymerase chain reaction (PCR) was performed, followed by capillary electrophoresis (CE). For PCR, fluorescently tagged PCR primers were designed (Integrated DNA Technologies (IDT), Coralville, IA, USA) to amplify 17 regions corresponding to 10 genomic loci (Figure 1A,B). PCR was carried out for those 17 regions using GeneAmp10X PCR Gold Buffer & MgCl_2_, dNTPs, and AmpliTaq Gold DNA Polymerase (Thermo Fisher Scientific) with specific conditions as described in Figure 1.

Loci-specific PCR products were mixed with HiDi Formamide (Thermo Fisher Scientific) to prepare the sample for CE. Samples were then loaded onto the ABI3730 genetic analyzer (Thermo Fisher Scientific) on a 36 cm capillary with POP7 polymer and GeneScan^®^ 400HD ROX size standard (Thermo Fisher Scientific). CE was performed at 60 °C with an injection voltage of 2.5 kV and an injection time of 30 s. Data analysis was performed using GeneMapper™ v4.0 software (Thermo Fisher Scientific).

### 2.4. Mutation Detection by Sanger Sequencing

**Figure 2:**
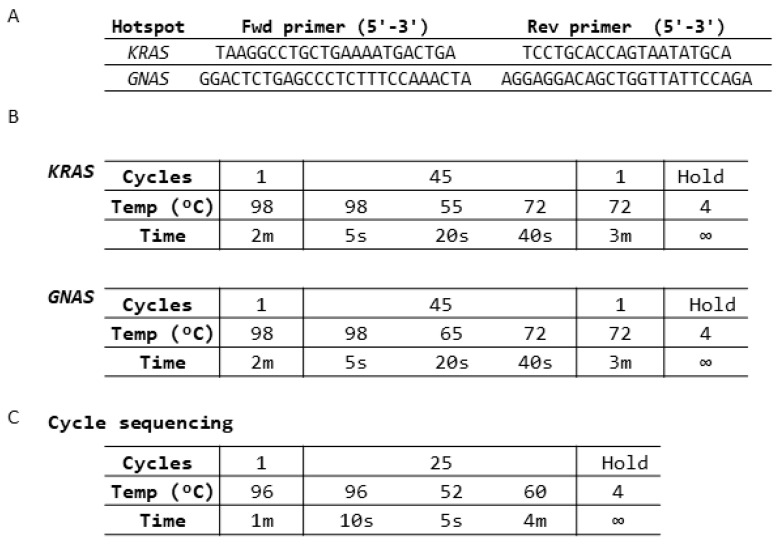
Design strategy for SNP detection in the *KRAS* and *GNAS* hotspot regions. (**A**) Primer sequences for mutation detection. (**B**) PCR conditions for the specific amplification of hotspot regions in the *KRAS* and *GNAS* genes. (**C**) Cycle sequencing conditions for Sanger sequencing.

Mutations in hotspot regions of the *KRAS* and *GNAS* genes were analyzed by cycle sequencing and dideoxy chain termination. Targeted regions in the *KRAS* and *GNAS* genes were PCR-amplified by specific primers using the KAPA SYBR FAST kit (Roche, Basel, Switzerland) with specific conditions as described in Figure 2.

The resulting PCR products were purified using SOPE resin (EdgeBio, San Jose, CA, USA) and Performa Edge Gel Filtration cartridges (EdgeBio) to remove PCR enzymes and buffers. Cycle sequencing PCR was carried out using the *KRAS*/*GNAS* PCR product with sequencing-specific primers (IDT) and Big Dye Terminator v1.1 (Thermo Fisher Scientific) to incorporate labeled nucleotides with specific conditions (Figure 2C). This reaction was purified using SOPE resin/Performa Edge Gel filtration to remove unincorporated dye-labeled dNTPs. HiDi Formamide (Applied Biosystems, Foster City, CA, USA) was added to the sequencing samples, followed by base calling on the ABI3730 genetic analyzer (Thermo Fisher Scientific). Sequencing data were analyzed using GeneScan^®^ v5.2 analysis software (Thermo Fisher Scientific).

### 2.5. Orthogonal Methods

Two methods were used to orthogonally assess the panel developed in this study. These include long-read third-generation sequencing (TGS) to verify the accurate detection of STR loci and short-read next-generation sequencing (NGS) to verify mutation detection.

#### 2.5.1. TGS—PacBio HiFi Sequencing

Long-read TGS was performed by the Institute for Genome Sciences (IGS) at the University of Maryland School of Medicine. LOH region-specific PCR was performed using the PCR conditions listed in Figure 1. PCR amplicons were converted to NGS libraries using the PacBio HiFi Library Preparation kit (PacBio), followed by PacBio Sequel II/IIe SMRT Cell 8M sequencing (Pacific Biosciences, Menlo Park, CA, USA). TGS reads were aligned to the Hg19 genome using BWA, followed by genotyping using TRcaller [56,57].

#### 2.5.2. NGS—Illumina Sequencing

Short-read NGS was performed by the Interpace Diagnostics clinical laboratory using clinical standard operating procedures as described previously [58,59]. Briefly, hotspot regions of *KRAS* and *GNAS* genes were amplified using specific primers to create barcoded NGS libraries, followed by 2 × 150 bp sequencing on the Illumina MiSeq sequencer (Illumina, San Diego, CA, USA). NGS reads were aligned to the Hg19 genome using Burrows-Wheeler Aligner (BWA) v0.7.17, and variants were called using a customized Genome Analysis Toolkit (GATK) v1.6 pipeline [57,58,60].

## 3. Results

### 3.1. Design and Specific Detection of LOH Regions and Hotspot Mutations

#### 3.1.1. Detection of LOH at 17 STR Regions

Primers were designed to amplify 17 highly polymorphic short tandem repeat (STR) regions corresponding to ten tumor suppressor genomic loci for measuring the LOH. The primers were designed to include a flank region on either side of the STR region, allowing for the amplification of any expansions/deletions resulting from STR polymorphism. The genomic coordinates and expected amplicon size based on the coordinates are listed in the Hg19 genome (Table 1). 

The primer design included the attachment of a fluorophore (HEX or FAM) to one of the primers, enabling the measurement of relative fluorescence units (RFUs) from the PCR amplicons using capillary electrophoresis (Figure 1A–C).

#### 3.1.2. Detection of Mutations by Sanger Sequencing

Primers were designed for hotspot regions in the *KRAS* (codon 12 and 13) and *GNAS* (codon 201) genes to detect well-characterized driver mutations in pancreatic cancer by Sanger sequencing. The primers, PCR, and cycle sequencing conditions for Sanger sequencing are shown in Figure 2A–C, respectively.

#### 3.1.3. Orthogonal Confirmation of LOH Detection

**Figure 3:**
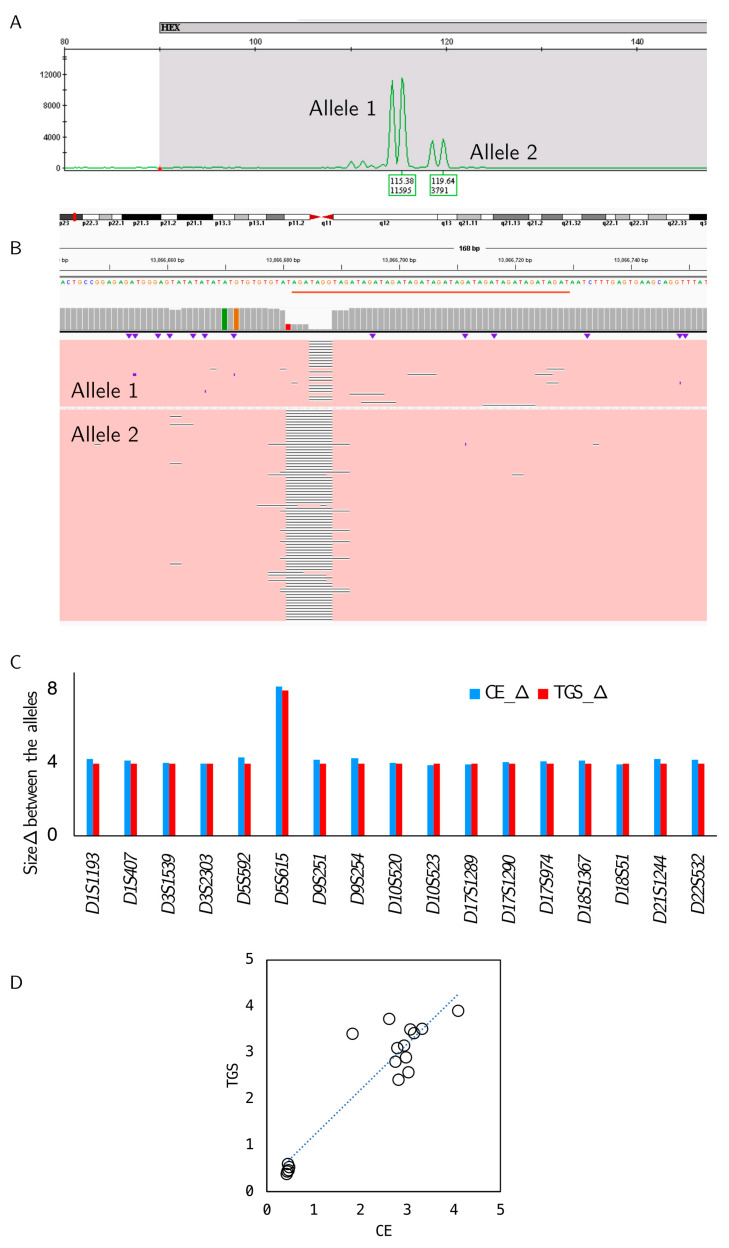
Concordance between the CE and TGS results. (**A**) Representative images show the capillary electropherogram for the *D9S254* STR region for a sample with a 3:1 allele ratio. (**B**) Corresponding IGV snapshot of the TGS alignment. (**C**) Concordance was observed for the allele sizes determined by both CE (blue) and TGS (red) for all 17 panel regions. (**D**) Allelic ratios detected by the 2 platforms (TGS versus CE) for all 17 regions were similar.

Specific detection of the LOH was orthogonally confirmed by long-read TGS. Well-categorized mixed blood control samples that mimic a positive allele imbalance, detected as positive for LOH by CE (*n* = 17, one sample per locus), were analyzed by TGS. A representative CE electropherogram and IGV snapshot of the TGS alignment shows the specific detection of the *D9S254* region (Figure 3A,B). High-resolution images are shown in Supplementary Figures S1 and S2. Comparison of the CE and TGS data showed the specific detection of all 17 genomic loci and a 100% agreement in allele sizes detected by two of the platforms for all 17 samples (Figure 3C), with a 0.83 coefficient of determination (R^2^) of the allele ratios called between the platforms (Figure 3D).

#### 3.1.4. Orthogonal Confirmation of Mutation Detection

**Figure 4:**
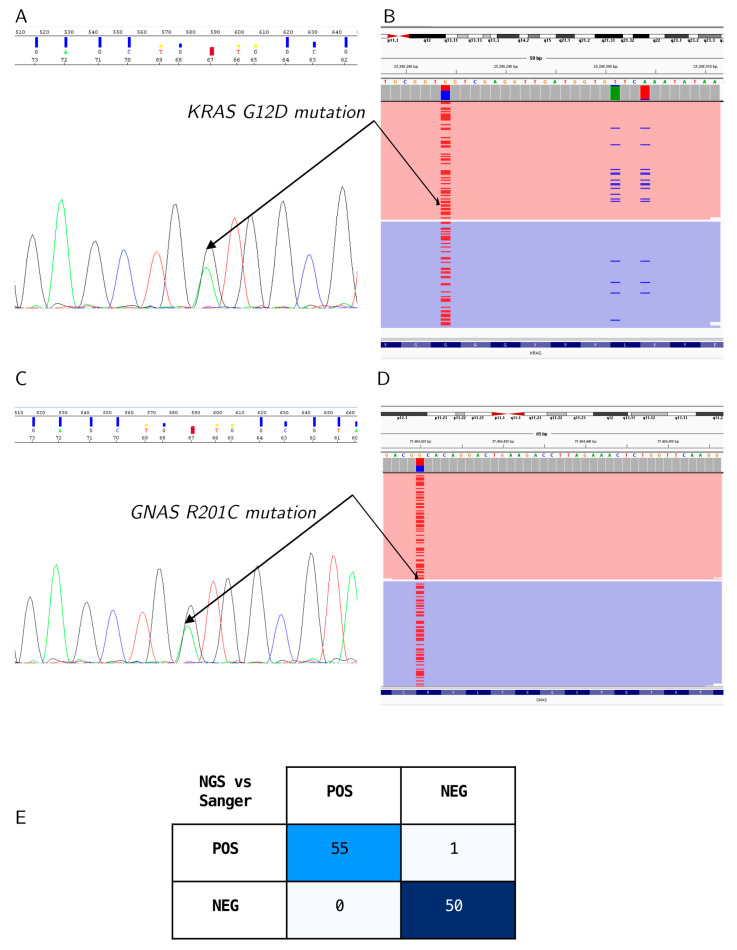
Concordance between Sanger sequencing and NGS. (**A**) Representative Sanger sequencing chromatogram for a sample with a *KRAS_G12D* mutation, and (**B**) the corresponding IGV snapshot of the *KRAS* mutation detected by NGS. (**C**) Representative Sanger chromatogram for a sample with a *GNAS*_*R201C* mutation, and (**D**) the corresponding IGV snapshot of the *GNAS* mutation detected by NGS. (**E**) A 2 × 2 contingency table shows a comparison of the Sanger and NGS results with 99% agreement between the platforms.

The specific detection of single-nucleotide polymorphisms (SNPs) in the *KRAS* and *GNAS* hotspot mutations was orthogonally confirmed by analyzing samples (*n* = 106, 56 positive and 50 negative) by short-read NGS. Capillary electropherograms (Figure 4A,B) and IGV snapshots (Figure 4C,D) show the alignment of the *KRAS* and *GNAS* amplicons by NGS (Figure 3A,B) and matching mutation calls. High-resolution images of the CE and NGS data are shown in Supplementary Figures S3–S6. A 99% agreement in calls was observed with one sample with a *KRAS* mutation missed by Sanger sequencing, where it was present below the Sanger Sequencing detection sensitivity of 10% variant allele frequency (% VAF) (Figure 4E).

### 3.2. Accuracy

**Figure 5:**
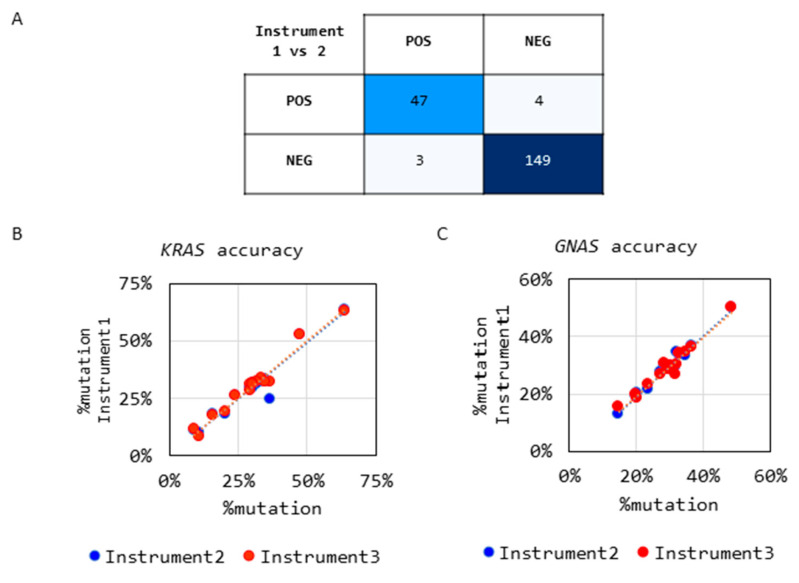
Accuracy of the LOH and mutation detection. (**A**) A 2 × 2 contingency table shows the accuracy of LOH detection by two independent PCR instruments: ABI9700 and ABI ProFlex thermocyclers, followed by an analysis with ABI3730 CE instruments. Correlation plot showing the detection accuracy of (**B**) *KRAS* and (**C**) *GNAS* mutations detected with 3 instruments.

The accuracy of LOH detection was assessed by PCR amplifying DNA from pancreatic cyst fluid samples (*n* = 203) on two different instruments. The samples were independently amplified on the ABI9700 and ABI ProFlex thermal cyclers followed by CE on two separate ABI3730 instruments. The resulting calls were compared, and 97% agreement was observed between the two platforms (Figure 5A).

Accuracy of the mutation detection was analyzed by PCR amplifying DNA from pancreatic cyst fluid by analyzing 30 samples (15 negative and 15 positive) each for the *KRAS* and *GNAS* mutations, followed by sequencing on an ABI 3130 genetic analyzer and two ABI 3730 genetic analyzer instruments. A 100% concordance in the negative mutation calls for both *KRAS* and *GNAS* across all three instruments was observed. Also, a 100% agreement was observed in the positive samples, with a correlation coefficient of >0.9 for the %VAF detected (Figure 5B,C).

### 3.3. Reproducibility

**Figure 6:**
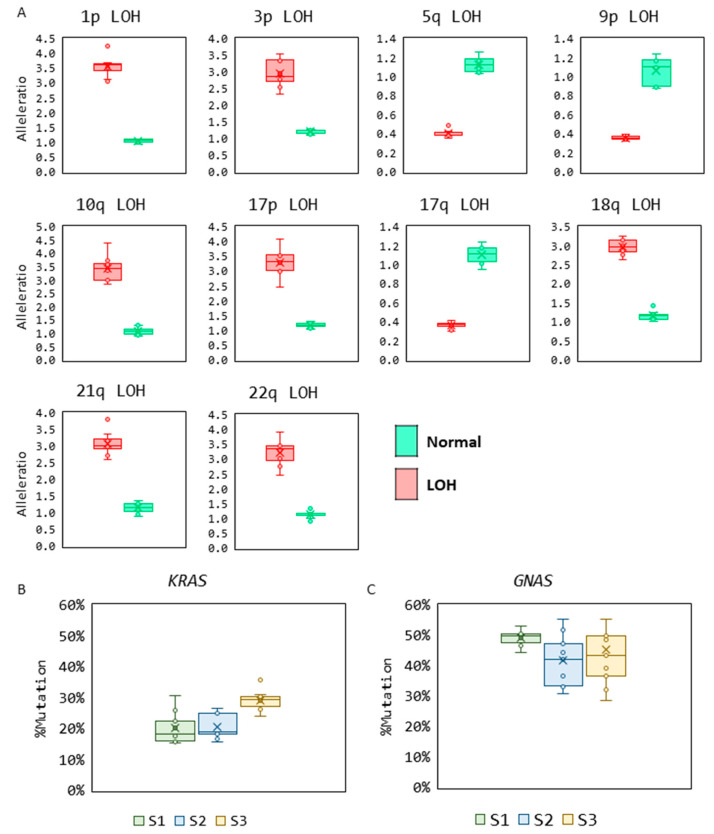
(**A**) Reproducibility of LOH and mutation detection. (**A**) Box and whisker plots show allele ratios and LOH detection at 10 genomic loci for paired samples—normal (green) and LOH sample (red) (*n* = 8). (**B**) Box and whisker plots show the reproducibility in measurements of % mutation in the *KRAS* mutant samples (*n* = 9). (**C**) Box and whisker plots show the reproducibility in measurements of % mutation in the *GNAS* mutant samples (*n* = 9).

The reproducibility of LOH detection was analyzed by testing 10 sample pairs corresponding to 10 loci analyzed for allelic imbalance. A heterozygous normal sample, where both alleles in the sample are present in a 1:1 ratio, and their matching patient sample with LOH where the two alleles present were in a 3:1 or 1:3 ratio, were analyzed across eight replicates (*n* = 8). An allele ratio of 1:1 was detected in all the normal samples, and LOH was detected near the expected allele ratio of either 3:1 or 1:3 in all the samples analyzed (Figure 6A).

The reproducibility of mutation detection was assessed by testing three positive samples each for the *KRAS* and *GNAS* mutations across three different instruments in triplicate (*n* = 9). The samples were detected consistently at the same % mutation across all replicates (Figure 6B).

To further demonstrate the reproducibility of the assay in a clinical setting, stringent quality control measures were in place, and the performance of the assay controls was routinely monitored. Plots show the performance of the controls from 1 month of clinical testing, which indicate the controls measured fell within ±2 standard deviations for all LOH loci, *KRAS,* and *GNAS* mutations analyzed (Supplementary Figures S7–S9).

### 3.4. Limit of Detection

**Figure 7:**
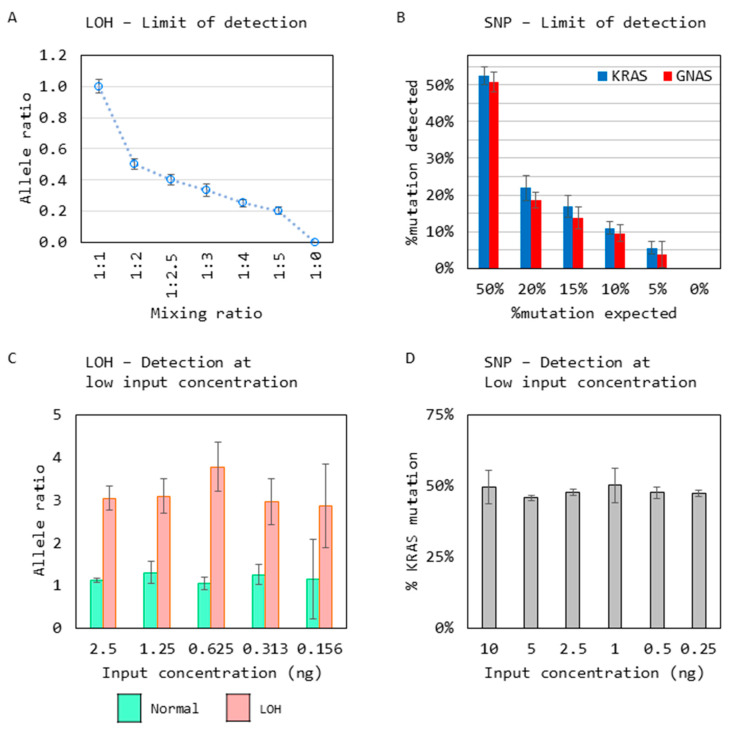
Limit of detection analysis for (**A**) shows a change in allelic imbalance detected by analyzing mixed samples with an increasing allelic imbalance at the *D5S615* STR region (*n* = 23). (**B**) *KRAS* and *GNAS* mutations were detected reliably at 10% VAF when samples positive for *KRAS* and *GNAS* mutations, respectively, were mixed into a negative sample to create samples ranging from 50% to 5% VAF and tested along with a negative sample (*n* = 21). (**C**) Reliable detection of the LOH at low-input concentrations (*n* = 8). (**D**) Reliable detection of mutations at low-input concentrations (*n* = 5). All graphs show the mean ± SD from replicates.

The limit of detection (LOD) for the LOH analysis was carried out by mixing two samples homozygous for the *D5S615* STR to create a range of samples reflecting an allelic imbalance, followed by CE analysis (*n* = 23). An allelic imbalance was accurately detected at the expected mixing ratio, reflecting a shift in the allele balance of 1:1 to 5:1 (Figure 7A).

For *KRAS* and *GNAS* mutation detection LOD, a mutation-positive sample was diluted into a mutation-negative sample to create samples across a range of % VAF followed by Sanger sequencing (*n* = 21). Both *KRAS* and *GNAS* mutations were detected successfully at 5% VAF (Figure 7B).

Finally, the minimum input concentration needed for the detection of LOH and mutations was assessed, as it is critical for the analysis of low-volume samples typically obtained from pancreatic fine-needle aspirates.

For the LOH input requirement, a sample positive for imbalance at the *D5S615* STR region was analyzed with its matching normal sample at various input concentrations ranging from 2 ng to 0.156 ng, where the sample allele ratios were detected consistently across all inputs (*n* = 8) (Figure 7C). Similarly, for mutation detection, a *KRAS* mutant sample was sequenced with varying input concentrations ranging from 10 ng to 0.25 ng, and *KRAS* mutation was detected successfully across all inputs (*n* = 5) (Figure 7D).

## 4. Discussion

Highly viscous pancreatic fine-needle aspirates are challenging samples to acquire, leading to low sample volume. The collected cell-free DNA is highly damaged due to pancreatic enzymatic activity, yet these samples provide a great opportunity to ascertain the progression of pancreatic disease. Consequently, developing robust analytical methods to reliably detect pathogenic variants for disease progression is critical [13].

Among these molecular changes are the loss of heterozygosity (LOH), the second of the two-step tumor suppressor inactivation described by Knudson [36]. Many growth-controlling genes exist within the human genome, often located in close chromosomal proximity to tumor suppressor-associated genes. Neoplastic progression towards malignant transformation and metastasis is caused by deletions of these genes [33].

STRs or microsatellites provide excellent biomarkers for analyzing the pathogenesis in pancreatic cysts, as allelic imbalances reflect chromosomal and microsatellite instability (MSI), which induces disease. A deficiency in the mismatch repair system produces MSIs but also increases the genetic mutation rate, particularly the sequence polymorphisms (SNPs). Most SNPs are not pathogenic; however, MSIs and chromosomal instability are strongly correlated with disease [61,62].

Of the 17 STR regions analyzed in this assay, the amplicon sizes range from 125 bp to 360 bp (Table 1), with a potential for expansion of the amplicon based on the number of repeats present. The unique size range of these STRs prohibits NGS from sequencing longer repeats in a single read due to the maximum NGS read length of 300 bp. For shorter repeats, sequencing is possible but requires the presence of sequences flanking the STRs on both sides, enabling reliable mapping for detection. However, this approach can be affected by both the STR sequence length variation and expansion alterations.

TGS has enough sequencing length per read for STR diagnostic viability. However, the library preparation methods recommend larger fragments (10 kb to 20 kb) and include size selection methods to remove shorter fragments [63]. Nonetheless, we sequenced the same amplicons generated by the CE assay by adding PacBio adapters without size selection and sequenced short amplicons on a long-read sequencer. With this approach, we orthogonally confirmed the CE STR analysis.

Although STR detection with TGS is possible and more suitable compared to NGS, longer-read sequencing is not cost-effective for this application at scale. The STR range of 125bp to 360 bp wastes up to 75% of the sequencing depth available [64]. CE can provide the same result with less complicated informatics, an easier workflow, and from an instrument more readily available in the community than TGS. We show that sequencing unique STRs that are too long for short-read and too short for long-read sequencing are best analyzed by CE for detecting allelic imbalances in our application.

As CE is ubiquitous in the community, we also demonstrated a method to analyze cognized oncogene SNPs using the gold standard—Sanger sequencing and capillary electrophoresis for genotyping. Validating the NGS data confirmed the detection of oncogenic driver SNPs. There are many advantages to NGS, including an increase in the number of SNPs per analysis/sample, but for this application, it is a disadvantage, as NGS variants can scale up but cannot scale down. For our application, it is not cost-effective to measure a few SNPs with NGS and, from a technical perspective, leads to a high sequencing duplication rate. Unless unique molecular indices (UMIs) are employed, PCR duplicates and optical duplicates from sequencing increase greatly and produce artificial SNPs; even with UMIs, the cost and informatic burden are greater than the gold standard of Sanger sequencing [65]. Lastly, the sensitivity advantage provided by NGS would not be actionable for *GNAS* and *KRAS* alone, as these occur early in the disease progression and are present in benign cysts as well.

In summary, the unique amplicon size/read length (200 bp to 500 bp) required for the targeted STRs and low number of SNPs present a unique set of analytical challenges. Although new methods and instrumentation for monitoring allelic imbalances and SNPs exist, the practicality and utility of capillary electrophoresis and Sanger sequencing remain highly effective and suitable in a clinical setting. Furthermore, multiple clinical studies have applied this technique for analyzing allelic imbalances to determine cancer aggressiveness and progression [12]. Similarly, Sanger sequencing continues to be the gold standard for DNA sequencing. It remains a cost-effective method for analyzing single genes or smaller mutational panels compared to NGS [34,59,61].

## 5. Conclusions

Pancreatic cancer has a low incidence of occurrence, a high incidence of cystic precursors, high morbidity and mortality associated with surgical intervention, and a low 5-year survival rate of 12%. This creates a tremendous burden on practicing clinicians in terms of how to best monitor and treat this disease. A reliable and accurate diagnostic test is needed for a primarily older patient population affected by pancreatic cancer precursors, which are associated with a substantial number of unneeded surgeries.

Our data demonstrate that, using Sanger sequencing (the gold standard), we can detect mutations in hotspot regions of the *KRAS* (codons 12 and 13) and *GNAS* (codon 201) genes accurately and precisely. More importantly, utilizing CE, we can accurately and precisely detect allelic imbalances via STR ratio differences. The data demonstrate the suitability of CE and Sanger sequencing over NGS and TGS for this set of biomarkers.

Lastly, utilizing Sanger sequencing requires a single CE instrument instead of two instruments, reducing the cost of instruments, maintenance, and lab space. This methodology and instrumentation have applicability in economically mindful settings and low-resource environments, where the newest instrumentation and technologies are not always available. Taken together, these results further demonstrate the continuing applicability of CE instrumentation in a clinical setting. The use of these methodologies provides a strong addition to the pancreatic diagnostic community.

## Figures and Tables

**Table 1:** Loss of heterozygosity detection using a panel of 17 STR regions. Listed below are the regions of interest, primer sequences used for amplification, and the expected amplicon size based on the Hg19 genome sequence.

Locus	STR_ Region	Amplicon_ Size	Chr	Start	End	Fwd Primer (5′-3′)	Rev Primer (5′-3′)
1p	*D1S1193*	180	chr1	12577512	12577692	TCGGCGACATAGCCAGAC	CTTTGATCTAAGGATTACCTAC
1p	*D1S407*	150	chr1	14854518	14854668	CTGTGCTAACCACATGGAG	GGGATAGAAGGATTAGTAGTG
3p	*D3S1539*	180	chr3	1064448	1064628	CTCTTTCCATTACTCTCTCC	TTCTCCATCTATCTTTCTCTC
3p	*D3S2303*	360	chr3	17952254	17952614	TGCCTACATGTTAGTATCCC	CTCCAGAGCTTTGTTTTCAAC
5q	*D5S592*	150	chr5	119101810	119101960	GGTGTCAACAAAGTAATGTAAAG	TGGATACATATTGTTTTCTGCTG
5q	*D5S615*	330	chr5	125163290	125163620	GAGATAGGTAGGTAGGTAGG	TCCACAGTGGTAAGAACCAG
9p	*D9S251*	390	chr9	30819368	30819758	TGCATGTTTTATGTGCACTAAC	CAATACTTTTTAAGGCTTTGTAGG
9p	*D9S254*	120	chr9	126869098	126869218	TGGGTAATAACTGCCGGAGA	GAGGATAAACCTGCTTCACTCAA
10q	*D10S520*	180	chr10	96424526	96424706	CAGCCTATGCAACAGAACAAG	GTCCTTGTGAGAAACTGGATGC
10q	*D10S523*	150	chr10	87006333	87006483	GGTGGAGGTTGTGGTGA	AACTGGGCATTTGTCTTTC
17p	*D17S974*	180	chr17	10518750	10518930	AGCCTGGGTGAGAGTGAGAC	GCCATTGTTAACAGGTTGGTG
17p	*D17S1289*	330	chr17	10859282	10859612	GCATGGTCTTTTTCCATTCC	CTGCCTCTAAGCAGTCATTTAGA
17q	*D17S1290*	240	chr17	56331496	56331736	CAGAGCAAGACTGTCCA	ACCAGGTGTCTCATAAG
18q	*D18S51*	180	chr18	60948976	60949156	CTCTGAGTGACAAATTGAGACCTTG	ACTTCTCTGGTGTGTGGAGA
18q	*D18S1367*	330	chr18	64552139	64552469	TTGGTTCATCCAAACATGGTAAA	CGGTGCCCACTAATTTATACCACAC
21q	*D21S1244*	300	chr21	25269091	25269391	TCTTCTATCTCATATGTGTATC	GGAGGAACTTGAGGATGTG
22q	*D22S532*	125	chr22	46123159	46123284	CCTGGGCAACAGAGCGAG	GTCTGAGAAGATACTTGATATAG

## Data Availability

The data presented in this study are available upon request. The data are not publicly available due to proprietary considerations.

## Supplementary Materials

The following supporting information can be downloaded at: https://www.mdpi.com/article/10.3390/diagnostics14050514/s1, Figure S1: Representative image shows the capillary electropherogram for the *D9S254* STR region for a sample with two alleles presented in a 3:1 allele ratio. Figure S2: Corresponding IGV snapshot of the TGS alignment showing the *D9S254* region for the sample analyzed in Supplementary Figure S1 by capillary electrophoresis. Figure S3: Representative image of a Sanger sequencing chromatogram showing the detection of a *KRAS*_G12D mutation indicated by the arrow. Figure S4: Integrated genomics viewer (IGV) snapshot shows a representative image of the NGS alignment, and the *KRAS*_G12D (indicated by an arrow) mutation detected in the sample was also analyzed by Sanger sequencing, as shown in Supplementary Figure S3. Figure S5: Representative image of a Sanger sequencing chromatogram showing the detection of a *GNAS*_R201C mutation indicated by the arrow. Figure S6: Integrated genomics viewer (IGV) snapshot shows a representative image of the NGS alignment, and the *GNAS*_R201C (indicated by an arrow) mutation detected in the sample was also analyzed by Sanger sequencing, as shown in Supplementary Figure S5. Figure S7: Levy Jennings plot shows the reproducibility of *D5S615* LOH detection in a clinical setting for one month. A process control sample with an expected 3:1 allele ratio is detected with a mean 3.09 allele ratio between the alleles (*n* = 185). Red and green lines show ±2 standard deviations from the mean. Figure S8: Levy Jennings plot shows the reproducibility of *KRAS* mutation detection in a clinical setting for one month. A process control sample (Horizon) with an expected 50% mutation is detected with a mean of 53.4% (*n* = 41). Red and green lines show ±2 standard deviations from the mean. Figure S9: Levy Jennings plot shows the reproducibility of *GNAS* mutation detection in a clinical setting for one month. A process control sample (Horizon) with an expected 50% mutation is detected with a mean of 51.5% (*n* = 41). Red and green lines show ±2 standard deviations from the mean.
