# Supplementary material for: Analytical Validation of Loss of Heterozygosity and Mutation Detection in Pancreatic Fine-Needle Aspirates by Capillary Electrophoresis and Sanger Sequencing
Source: Diagnostics (Basel). 2024 Feb 28;14(5):514. doi: 10.3390/diagnostics14050514 (PMC10930882; doi:10.3390/diagnostics14050514)
Supplement: Supplementary file 1 [file diagnostics-14-00514-s001.zip › diagnostics-2834658-supplementary.pdf]

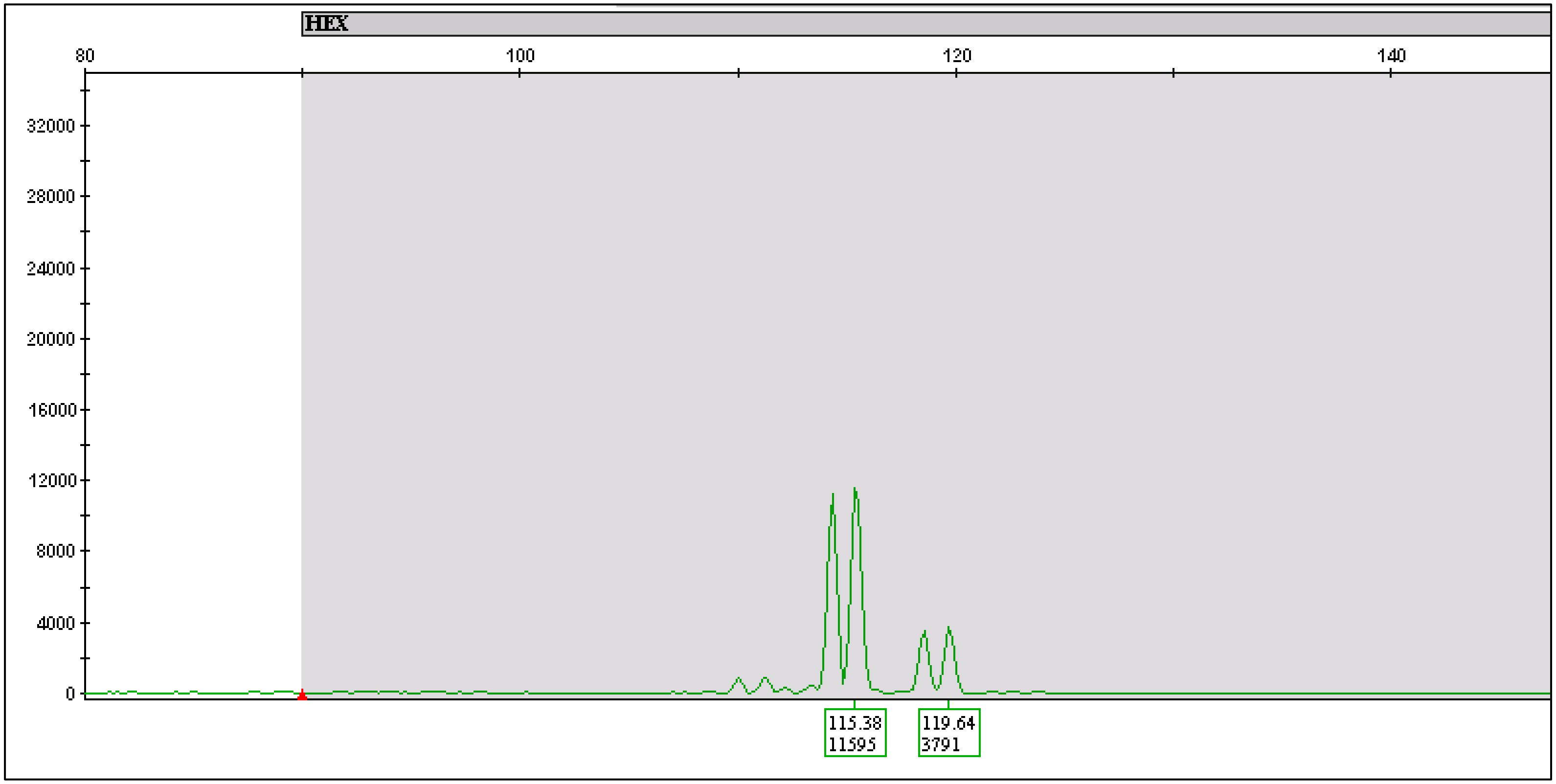

Supplementary Figure S1. Representative image shows the capillary electropherogram for the *D9S254* STR region for a sample with two alleles presented in a 3:1 allele ratio.

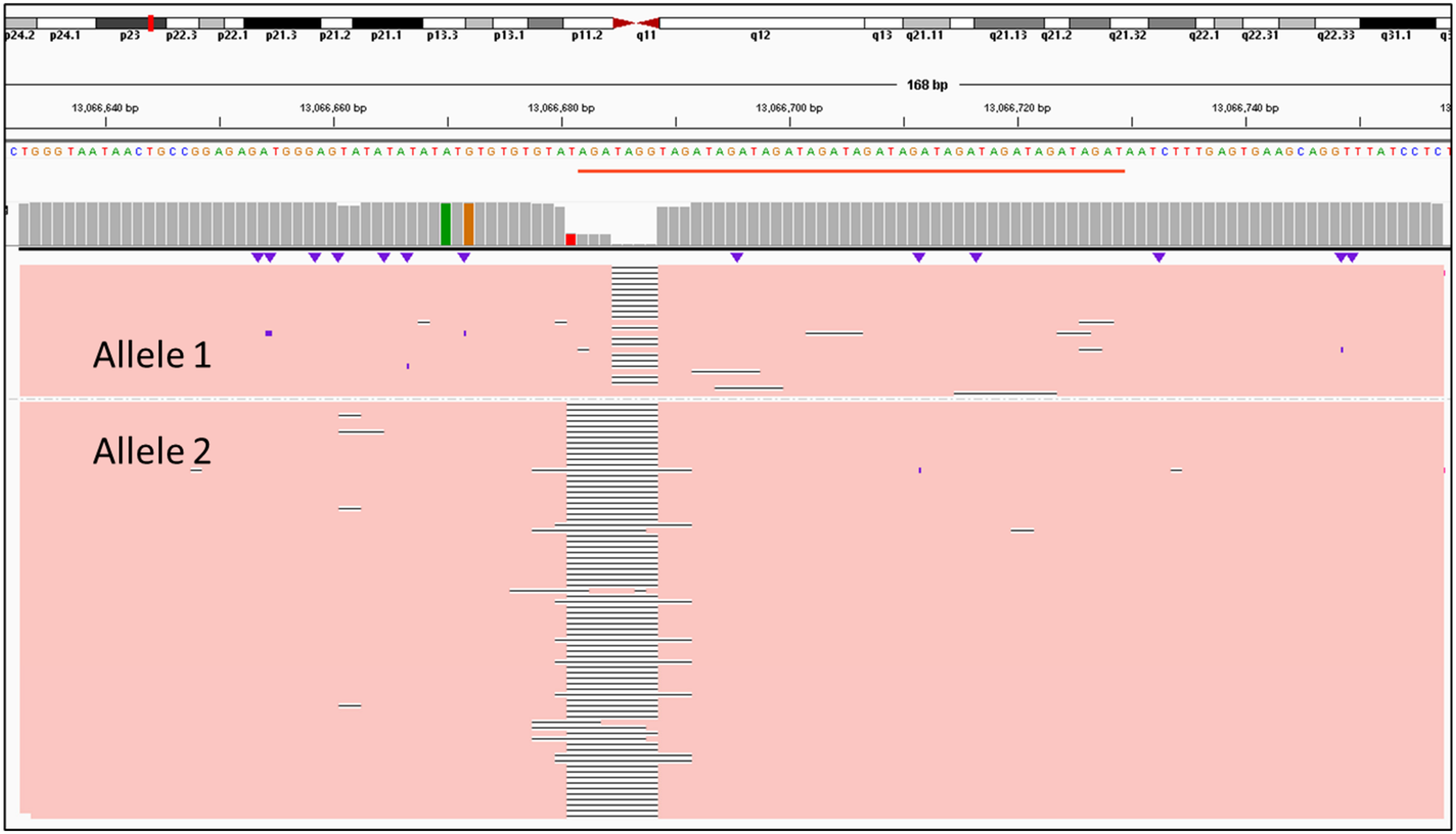

Supplementary Figure S2. Corresponding IGV snapshot of the TGS alignment showing the *D9S254* region for the sample analyzed in Supplementary Figure S1 by capillary electrophoresis.

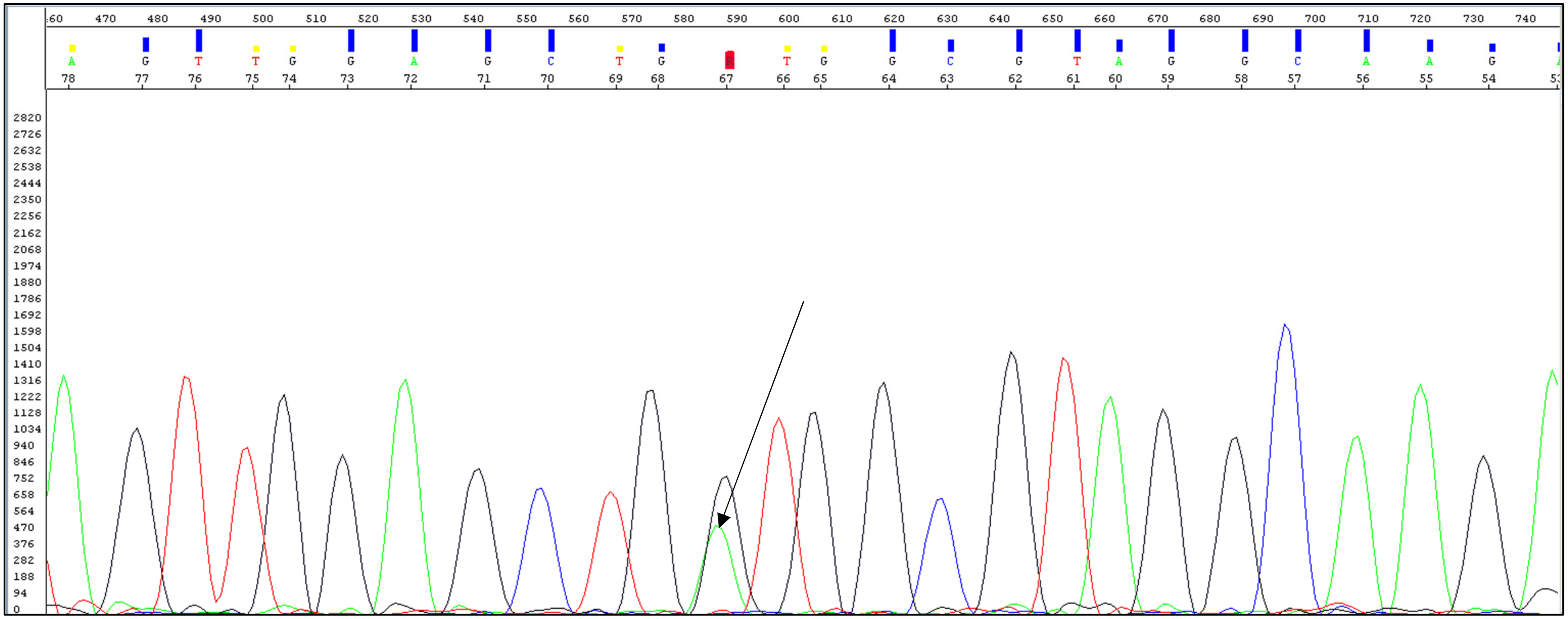

Supplementary Figure S3. Representative image of a Sanger sequencing chromatogram showing the detection of a *KRAS*_G12D mutation indicated by the arrow.

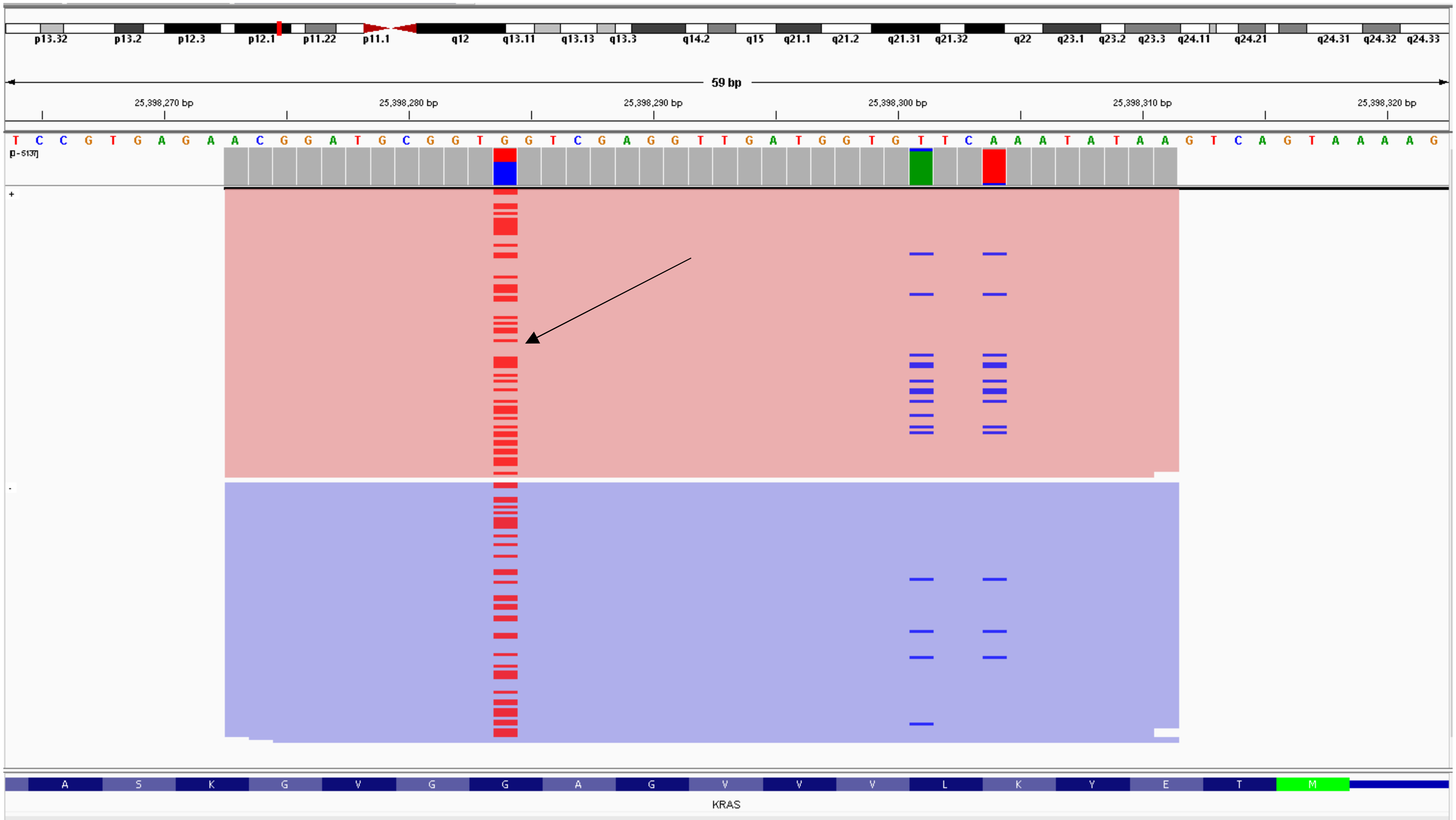

Supplementary Figure S4. Integrated genomics viewer (IGV) snapshot shows a representative image of the NGS alignment, and the *KRAS*_G12D (indicated by an arrow) mutation detected in the sample was also analyzed by Sanger sequencing, as shown in Supplementary Figure S3.

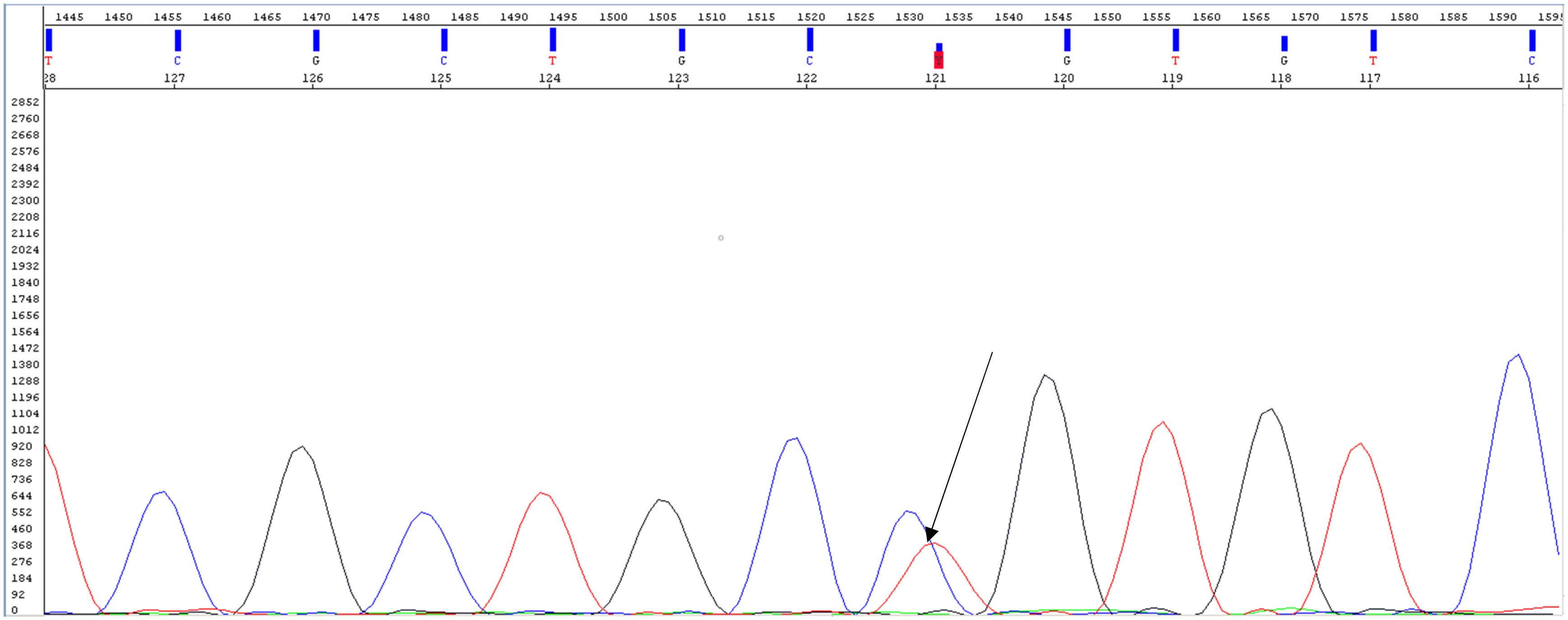

Supplementary Figure S5. Representative image of a Sanger sequencing chromatogram showing the detection of a *GNAS*_R201C mutation indicated by the arrow.

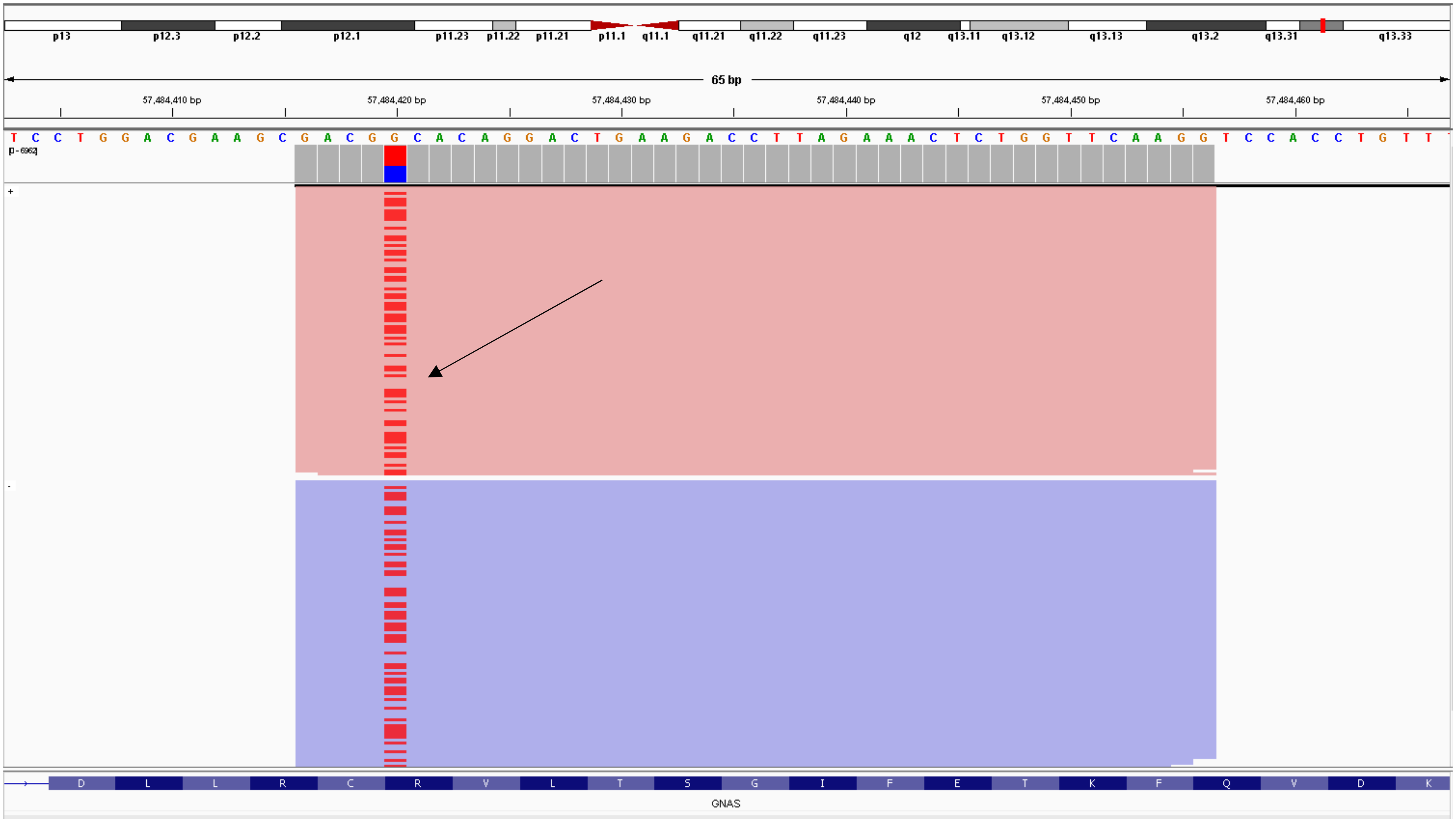

Supplementary Figure S6. Integrated genomics viewer (IGV) snapshot shows a representative image of the NGS alignment, and the *GNAS*_R201C (indicated by an arrow) mutation detected in the sample was also analyzed by Sanger sequencing, as shown in Supplementary Figure S5.

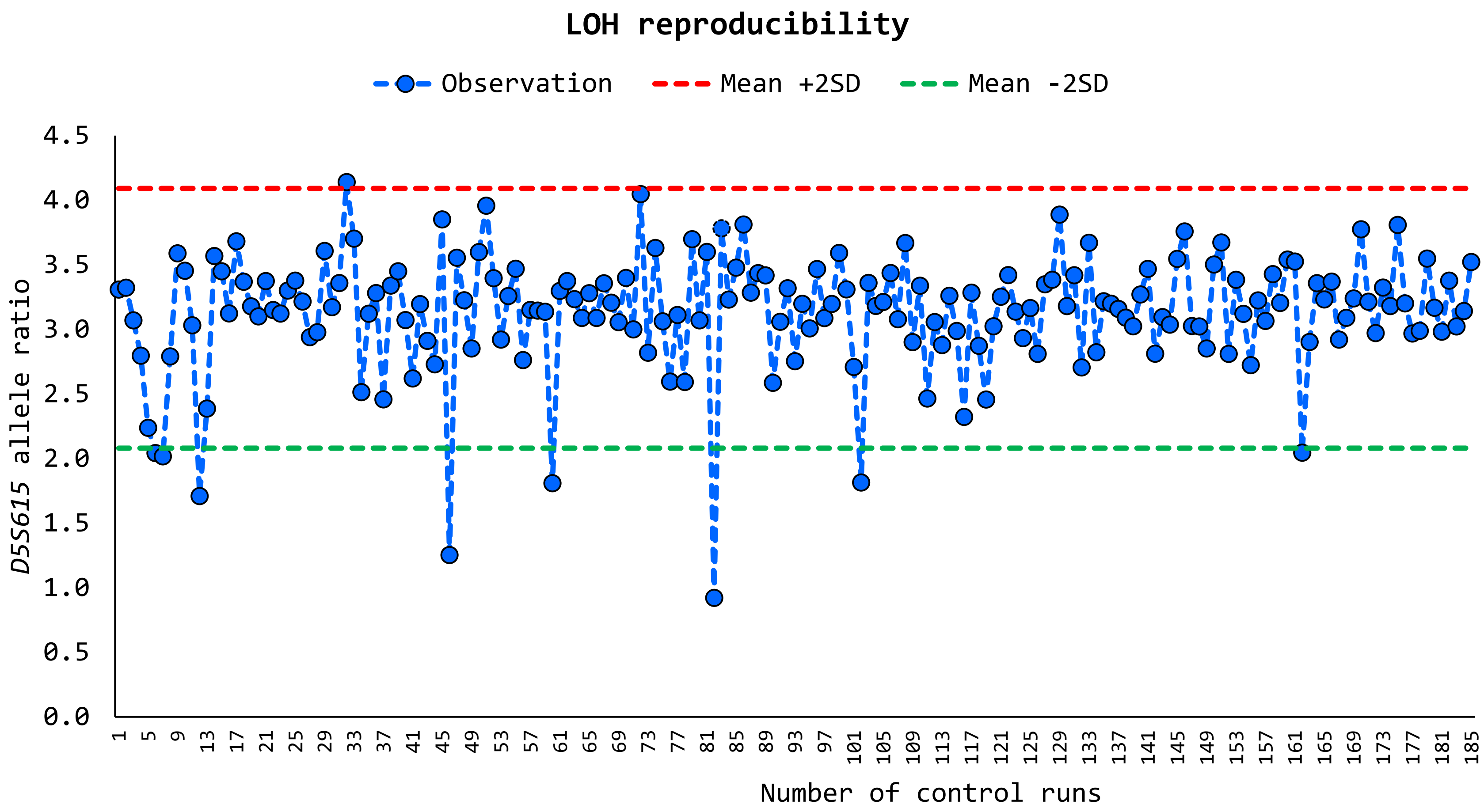

Supplementary Figure S7. Levy Jennings plot shows the reproducibility of *D5S615* LOH detection in a clinical setting for one month. A process control sample with an expected 3:1 allele ratio is detected with a mean 3.09 allele ratio between the alleles (*n* = 185). Red and green lines show ±2 standard deviations from the mean.

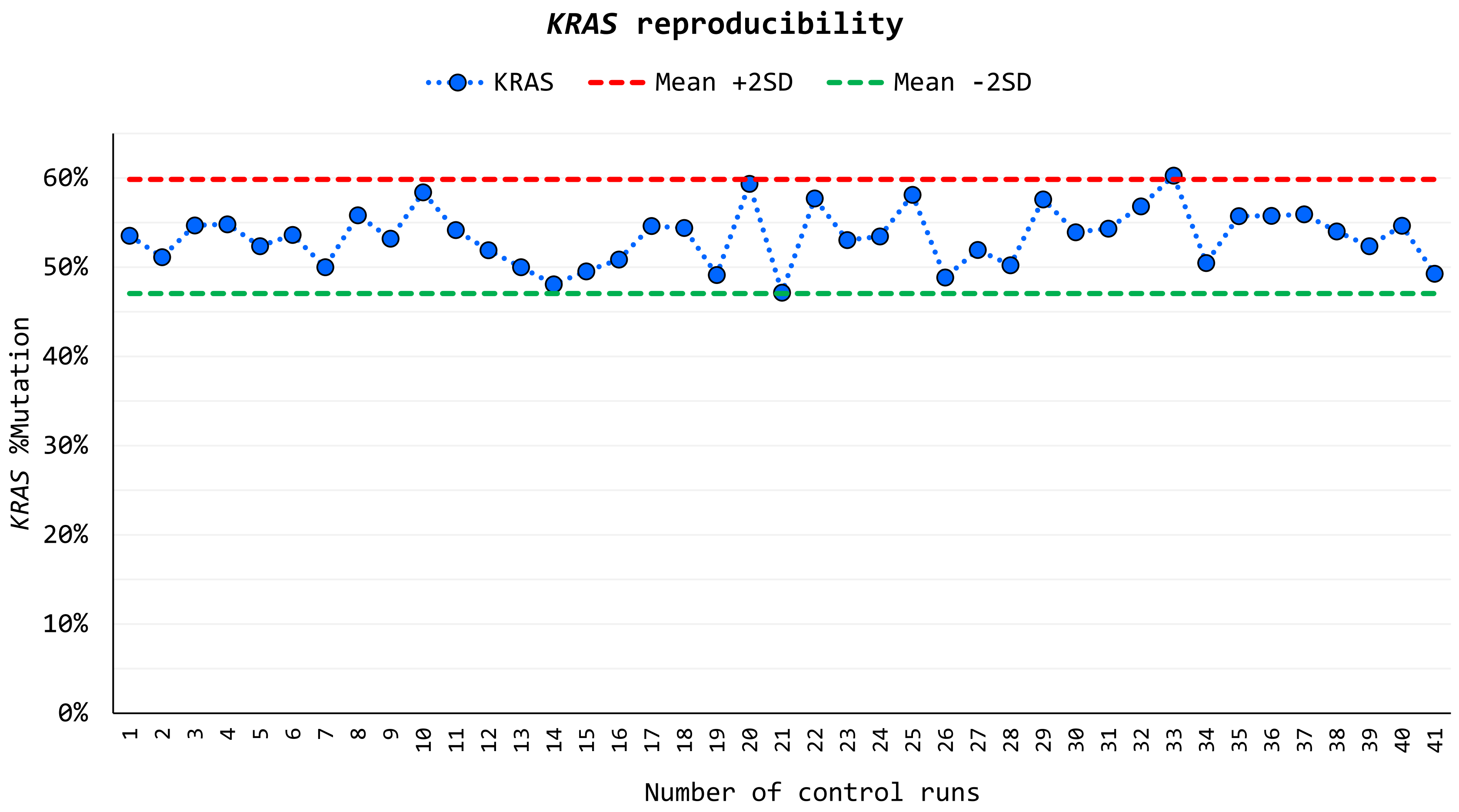

Supplementary Figure S8. Levy Jennings plot shows the reproducibility of *KRAS* mutation detection in a clinical setting for one month. A process control sample (Horizon) with an expected 50% mutation is detected with a mean of 53.4% (n = 41). Red and green lines show ±2 standard deviations from the mean.

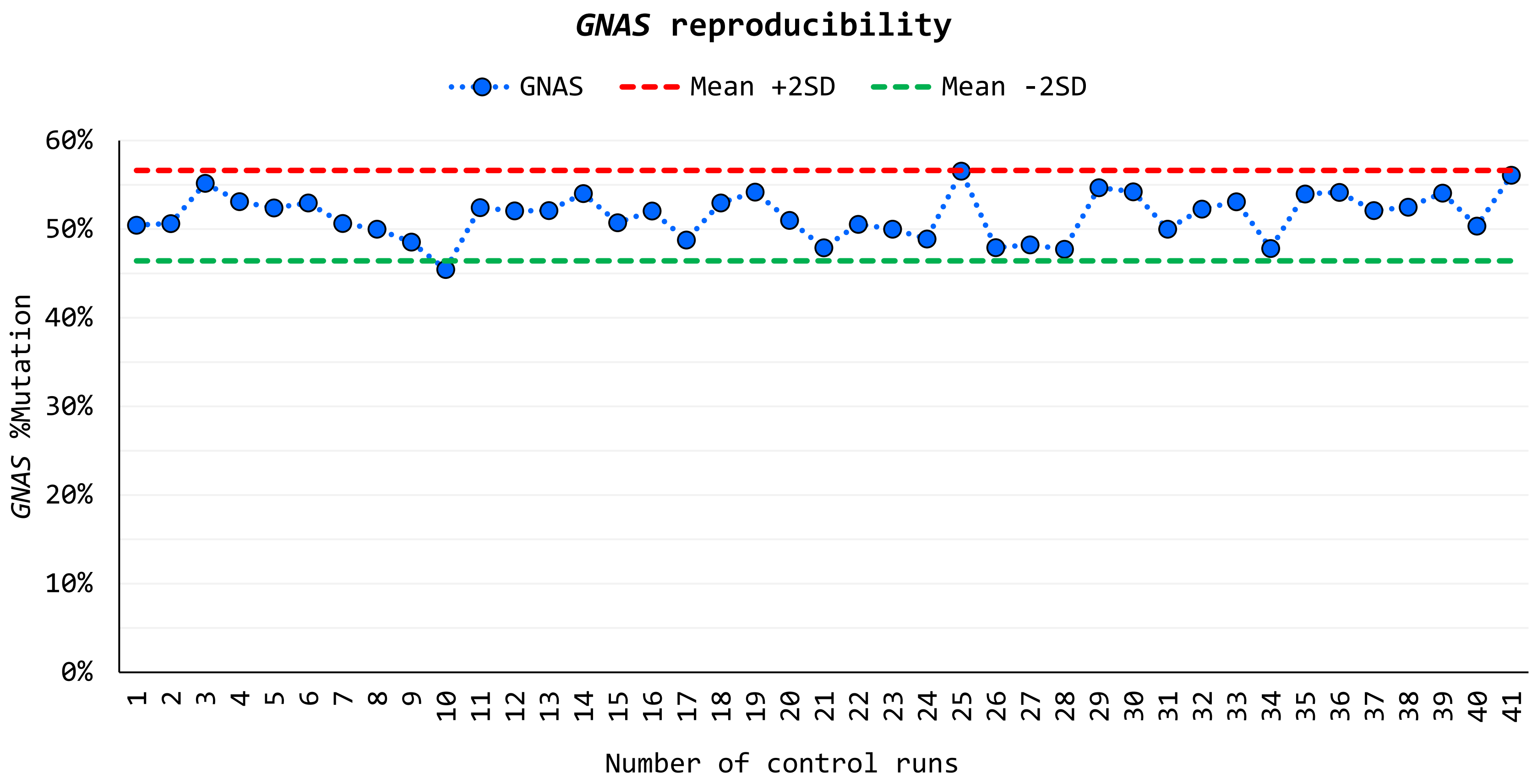

Supplementary Figure S9. Levy Jennings plot shows the reproducibility of *GNAS* mutation detection in a clinical setting for one month. A process control sample (Horizon) with an expected 50% mutation is detected with a mean of 51.5% (n = 41). Red and green lines show ±2 standard deviations from the mean.
